# Effects of Implicit Prosody and Semantic Bias on the Resolution of Ambiguous Chinese Phrases

**DOI:** 10.3389/fpsyg.2019.01308

**Published:** 2019-06-04

**Authors:** Miao Yu, Brandon Sommers, Yuxia Yin, Guoli Yan

**Affiliations:** ^1^Academy of Psychology and Behavior, Tianjin Normal University, Tianjin, China; ^2^School of International Exchange, Tianjin Foreign Studies University, Tianjin, China; ^3^Foreign Languages College, Tianjin Normal University, Tianjin, China

**Keywords:** prosodic boundary, semantic bias, ambiguity resolution, implicit prosody, silent reading, eye movement

## Abstract

By manipulating the location of prosodic boundary and the semantic bias of the ambiguous “V+N1+de+N2” phrase, which is composed of one verb (V), one noun (N1), one functional word (de), and another noun (N2), this study investigated how prosodic boundary and the semantic bias affect the processing of temporary ambiguous sentences formed by the ambiguous phrase “V+N1+de+N2” through an eye movement experiment. We found the effect of prosodic boundary in the late processing stage and observed an interaction between prosodic boundary and the semantic bias of ambiguous phrases as well. The participants required more time for fixation and more regressions occurred when the meaning of the ambiguous phrase guided by prosodic boundary was inconsistent with context, especially when the ambiguous phrase was biased to the narrative-object phrase. This result suggests that prosodic boundary affects the processing of temporal ambiguous sentences and is influenced by the semantic bias of the ambiguous phrase. These findings provide further evidence from Chinese that indicate that implicit prosody plays a general role in language comprehension.

## Introduction

Sentence processing is the process in which readers make use of a variety of information sources, such as lexical and syntactic information, semantic information, prosodic information etc., to extract syntactic structure and integrate meaning.

Research over the past 20 years has strongly suggested that prosody plays a very important role at multiple levels in the parsing process (Grabe et al., 1994; Snedeker and Trueswell, 2003; Watson and Gibson, 2005; Dahan, 2015).

As an important part of spoken language, prosodic information is a necessary means of language communication, which helps the listener to interpret syntactic structure and semantic information, so as to understand the utterance more clearly. Almost every aspect of prosody is conducive to the processing of spoken information. A vast amount of literature has confirmed prosodic information plays a very important role in speech production (e.g., Katz et al., 1996; Fodor, 1998; Steinhauer et al., 1999; Schafer et al., 2000; Clifton et al., 2002; Bradley et al., 2003; Fernández, 2007; Millotte et al., 2007; Li et al., 2010; Kentner, 2012; Tyler, 2013).

In addition, a wealth of perception studies have shown that listeners gain access to prosodic information and use prosodic boundary as cues to parse syntactic structure and resolve ambiguities in language processing (e.g., Marslen-Wilson et al., 1992; Nicol and Pickering, 1993; Beach et al., 1996; Speer et al., 1996; Schafer, 1997; Kjelgaard and Speer, 1999; Snedeker and Trueswell, 2003; Kraljic and Brennan, 2005; Schafer et al., 2005; Dede, 2010; Langus et al., 2012; Nakamura et al., 2012; Kurumada et al., 2014; Fromont et al., 2017).

In real life, the principal means by which people obtain information is through reading silently. Therefore, researchers are particularly interested in whether prosody has been at play in silent reading. In contrast to spoken language which possesses ample and overt prosody, written language does not contain the same abundance of prosodic information. Therefore, it is a challenge to study the effects of prosodic information during silent reading, but a large number of studies have shown that readers can activate prosodic representation even when silent reading. The phenomenon that sound representations are activated during silent reading is conventionally termed implicit prosody. According to the implicit prosody hypothesis (IPH), which was first proposed by Fodor (1998, 2002) and Bader (1998), a default prosodic contour is projected onto the stimulus in silent reading, and it may have an effect on syntactic ambiguity resolution.

The important effect of implicit prosody has been extended to many levels of language processing. Some studies have manifested that prosodic features at the lexical level contribute to processing during silent reading (Abramson and Goldinger, 1997; Ashby and Rayner, 2004; Lukatela et al., 2004; Ashby and Clifton, 2005; Ashby, 2006; Ashby and Martin, 2008; Huestegge, 2010; Breen and Clifton, 2011, 2013; Kentner, 2012; Luo et al., 2013, 2015; Gross et al., 2014; Yan et al., 2014; Yu and Ding, 2019), such as the influence of vowel length on word recognition (Abramson and Goldinger, 1997; Lukatela et al., 2004; Huestegge, 2010) and the effect that the number of lexical stress and stress patterns have on word comprehension (Ashby and Rayner, 2004; Ashby and Clifton, 2005; Ashby, 2006; Breen and Clifton, 2011, 2013).

A considerable amount of the research conducted on the effect of implicit prosody in language processing has been primarily focused on ambiguity resolution (e.g., Bader, 1998; Fodor, 1998, 2002; Fernández et al., 2003; Hirose, 2003; Jun, 2003; Swets et al., 2007; Hwang and Schafer, 2009; Traxler, 2009; Hwang and Steinhauer, 2011; McCurdy et al., 2013; Jun and Bishop, 2015; Drury et al., 2016; Webman-Shafran, 2018; Yao and Scheepers, 2018).

For instance, some studies found that implicit prosodic boundary, often marked by a pause, reduced the degree of difficulty in interpreting garden path sentences and facilitated the recovery from garden path sentences (Bader, 1998; Hwang and Steinhauer, 2011; Niikuni and Muramoto, 2014; Harris et al., 2016). Kentner (2012, 2017) have shown that readers can use implicit rhythmic information to dissolve ambiguous sentences during silent reading.

Regarding the effects of implicit prosodic boundary on syntactic ambiguity resolution, researchers have paid extensive attention to the attachment of relative clauses (RC) in which the RC can be interpreted as high attachment to the first noun phrase or low attachment to the second noun phrase.

Fodor (1998, 2002) has proposed the role of implicit prosody on the resolution of attachment ambiguities involving relative clauses. Fodor claims that cross-linguistic preference differences in whether the relative clause favors low attachment (attach to the immediately preceding noun) or high attachment (attach to the more distant noun) can be attributed to a cross-linguistic difference in the implicit prosody.

From then on, the effects of implicit prosody on resolution of attachment ambiguities has been proven by a wide range of cross-language studies, including English (Swets et al., 2007; Traxler, 2009; Jun and Bishop, 2015), Spanish (Fernández, 2003), German (Augurzky, 2006), Croatian (Lovrić, 2003), and Hebrew (Shaked, 2009). See also Breen (2014) for review discussion.

Some implicit prosody studies have focused on the extent to which the length of the sentence constituent influences attachment decisions (Selkirk, 2000; Fodor, 2002; Fernández et al., 2003; Hirose, 2003; Lovrić, 2003; Hwang and Schafer, 2009; Hwang and Steinhauer, 2011). The basic consensus of these studies is that the length of the relevant sentence constituent could influence the location of prosodic boundaries in sentences and thus alter the attachment decision.

Several other studies have examined the interaction between context and implicit prosody on syntactic ambiguity resolution in silent reading. McCurdy et al. (2013) investigated whether contextual bias influences the role of metrical structure in resolving syntactic ambiguity in silent reading. Their results indicate that implicit meter has a strong effect on parsing, one that seems irrelevant to higher-level constraints. Kentner and Vasishth (2016) also attested that discourse context and local linguistic rhythm conspire to guide the focus-structural analysis of ambiguous sentences. Yu and Yan (2015) found that prosodic boundary helps to dissolve the ambiguous structure when prosodic boundary is consistent with ongoing context. However, the prosodic boundary is not limited by the contextual bias, which can affect the resolution of ambiguity.

The aforementioned research shows that a growing body of evidence supports the IPH, especially the findings from cross-linguistic research supporting the role of implicit prosody in syntactic ambiguity resolution. However, there still exists some controversial issues that need to be further explored and discussed.

First and foremost, nearly all previous studies of implicit prosodic effect on syntactic ambiguity resolution have been centered on the attachment of RC. Further investigations on various constructions and in various languages are needed to test the IPH. There are a variety of ambiguous structures in Chinese. Some of them are different from those in western languages mentioned above. Does implicit prosody play the same role in ambiguity resolution in Chinese as in other languages? Does Chinese, which possesses different prosodic properties, present different effects of implicit prosody? There are few studies to date on implicit prosody in Chinese, therefore, the investigation on implicit prosody in Chinese is necessary to provide cross-language data to advance our understanding of implicit prosody.

Second, early studies did not specifically probe the time and the way in which the prosodic imformation is integrated with the linguistic information of the sentence (Cutler et al., 1997). In fact, to our knowledge, there has been rare dicussion of the relative time course of prosodic processing in sentence comprehension, particularly, there are few studies on the early exploitation of prosodic imformation in processing.

Third, although a host of research has shown that implicit prosody influences ambiguity resolution, there are few discussions exploring the issue of whether there are interactions between prosody and other factors. A few early studies revealed an interaction between prosody and the length of syntactic constituents or context on ambiguity resolution. With those factors aside, are there any other factors affecting prosody and how they interact with prosody? Those questions are worthy of further investigation.

In Chinese, one type of phrase, composed of one verb (V), one noun (N1), one functional word (de), and another noun (N2), is syntactically ambiguous. These phrases can be understood as a modifier-head structure (MHS), which is a phrase that contains a modifier and a head, or a narrative-object structure (NOS), which is a phrase that contains a predicate (often a verb) plus an object (often a noun). Generally speaking, the prosodic boundary can disambiguate the phrase. The prosodic boundary immediately following “V” motivates the whole “V+N1+de+N2” phrase to be parsed as NOS and corresponding meaning, while the prosodic boundary immediately following the functional word “de” motivates the entire “V+N1+de+N2” phrase to be parsed as MHS and corresponding meaning. In contrast to the attachment preference of RC in Western languages, the “V+N1+de+N2” phrase is more complex. Zhang (1998) mentioned that “V+N1+de+N2” phrases in Chinese have different semantic bias, which refers to a native speakers’ preference for one meaning over another meaning when interpreting ambiguous structures. Some of them are balanced between MHS and NOS. The others bias toward either MHS or NOS.

Previous production and perception studies found that the ambiguity of the “V+N1+de+N2” phrase can be effectively dissolved when the location of prosodic boundary is consistent with the syntactic structure (Wang et al., 2003; Yu, 2011), indicating that prosodic boundary provides active clues to dissolve the ambiguity. Li et al. (2010) found that prosodic boundaries can be used to direct syntactic ambiguity resolution during spoken discourse comprehension. Given this, does the prosodic boundary play a role in ambiguity resolution of the “V+N1+de+N2” phrase during silent reading? How and when do prosodic boundaries influence syntactic ambiguity resolution of the “V+N1+de+N2” phrase? Are there any interactions between prosodic boundaries and semantic biases?

To explore the above questions, two kinds of “V+N1+de+N2” ambiguous phrases, with narrative-object semantic bias and no semantic bias, were selected as target phrases and the two positions of prosodic boundary, after V and after “de,” were manipulated. The current study will employ the EyeLink 2000 eye tracker to explore the time course of prosodic processing and the interaction between semantic bias and prosodic boundary in disambiguating the “V+N1+de+N2” phrase in silent reading in Chinese. This syntactic structure has been rarely studied in silent reading hence it is our hope that this study will provide novel data in regard to the role of prosody in silent reading.

Eye movement experiments can reveal when and where processing difficulties arise and how readers solve them. For this reason, a large body of previous research has made use of eye movement experiments to explore the parsing process of ambiguous sentences.

Some research has found that when processing garden path sentences, readers will increase fixation duration on the disambiguating region and will regress from this region to the foregoing of the sentence (Frazier and Rayner, 1982; Rayner and Sereno, 1994; Kemper et al., 2004). The augmentation of fixation duration reflects the difficulty of processing; therefore, readers need to make regressions to do reanalysis (Meseguer et al., 2002).

According to the time in the course of processing, there are two categories of fixation: early measure which includes first fixation duration, first pass reading time, gaze duration etc.; and late measure which includes total fixation duration, regression path duration etc. (Liversedge et al., 1998; Rayner, 2009). The former reflects the parsing difficulties at the early stage while the latter reflects the reanalysis and reparsing of the information. Generally, the two measures, the probability of regression out and regression path duration, jointly reflect the readers’ reanalysis during cognitive processing.

Some studies observed processing difficulties on the disambiguating region even in the early stage of parsing the ambiguous construction. There was a longer first pass reading time on the disambiguating region for reduced relative clause sentences (Ferreira and Clifton, 1986; Trueswell et al., 1994). Additionally, Liversedge et al. (1998) also found that there was a longer first fixation duration and gaze duration on the disambiguating region for reduced relative clause sentences because reduced relative clause sentences are temporarily ambiguous as compared to unreduced sentences. Zhang and Shu (2002) investigated the mechanisms underlying the effect of referential discourse context on the parsing of Chinese ambiguous phrases. The influence of discourse context was found on the fixation duration of narrative-object structures and first pass reading time on the disambiguating region. These results suggested that the referential discourse context had influenced the parsing of the ambiguous phrases even at the very beginning of these phrases.

Eye movement experiments were also used to explore the readers’ reanalysis in parsing ambiguity. For example, Liversedge et al. (1998) found that there were regression path reading time and re-reading time on the region before the disambiguating region because reduced relative clause sentences are temporarily ambiguous as compared to unreduced sentences. The results showed that upon reaching the disambiguating region, readers made a regressive saccade indicating they took more time to examine the foregoing part of reduced relative clause sentences. Clifton et al. (2003) investigated the parsing of sentences with reduced relative construction. Their study found more regressions coming out of the disambiguating region when the verb followed the disambiguating phrase than when the verb directly followed the initial NP. This suggested that readers were prone to quickly read back to the preceding portion of the sentence immediately after interpreting the verb when that portion of the sentence contained a disambiguating phrase made up of “who/that was.”

Based on the research mentioned above, we expected to observe the effect of implicit prosody on dissolving ambiguity at the early stage of processing and to further understand whether implicit prosody plays a role immediately (in the local domain of the ambiguous sentence) or at the later stage of processing. In line with the experimental manipulation of the current study, we expected to find that subjects would increase fixations and regressions in the disambiguating region when the meaning guided by implicit prosody was inconsistent with the contextual meaning.

## Materials and Methods

### Participants

Forty native Chinese speakers (aged between 17 and 19 years, 25 females) participated in the experiment. All were freshmen at Tianjin Normal University, with normal or corrected-to-normal vision.

### Materials and Design

This study originally chose 200 “V+N1+de+N2” ambiguous phrases from some previous literature (Zhang, 1998; You, 2002; Zou and Ma, 2007; Li et al., 2010). Semantic bias of those 200 ambiguous phrases were rated on a seven-point scale (where a score of 1 was narrative – object bias, and a score of 5 was modifier-head bias) by 30 Chinese college students. Finally, 36 “V+N1+de+N2” ambiguous phrases with narrative-object bias (*M* = 3.18, *SD* = 0.22) and 36 balanced “V+N1+de+N2” ambiguous phrases (*M* = 4.01, *SD* = 0.18) were determined (see Supplementary Materials).

Four types of sentences were created by making use of the blank space to mark the prosodic boundary, each containing 36 sentences with an ambiguous phrase of “V+N1+de+N2” and a different prosodic boundary location. An example of a narrative-object biased ambiguous “V+N1+de+N2” phrase has been provided below (see Table 1).

**Table 1:** Example Chinese stimuli with literal translations from different conditions.

Prosodic boundary	Example
After “v”	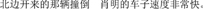
	Coming from the north that (ran into Xiao Ming’s car) was driving very fast.
After “de”	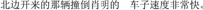
	Coming from the north that (the car which ran into Xiao Ming) was driving very fast.
Translation:	The car that was coming from the north which ran into Xiao Ming was driving very fast.


The naturalness and acceptability of sentences were rated on a five-point scale (1 being very unnatural, and 5 being very natural) by 80 Chinese students from Tianjin Normal University. They did not participate in the eye tracking experiment. The mean acceptability and naturalness scores of the sentences containing narrative-object biased “V+N1+de+N2” phrases were 4.20 (*SD* = 0.32) and 4.07 (*SD* = 0.38), whereas the mean naturalness and acceptability scores of the sentences containing balanced “V+N1+de+N2” phrases were 4.06 (*SD* = 0.31) and 4.15 (*SD* = 0.35). The length of the sentences ranged from 18 to 24 Chinese characters (*M* = 20.75 characters, *SD* = 2.01). The instructions of the ratings are presented in the Supplementary Material.

The experimental materials were divided into four lists. Each list contained 36 experimental sentences, counterbalanced using a Latin square. In addition, another 72 filler sentences were filled in each list. All sentences were presented randomly in a blocked format. Previous studies have reported very robust effects of implicit prosody in sentence processing, such as Wijnen (2004), Traxler (2009), and Kentner (2012). The average effect sizes presented in their studies were approximately 0.91, 0.63, and 1.80, respectively. Similar to our current study, Traxler (2009) also investigated the effect of implicit prosodic boundary on syntactic ambiguity resolution, therefore, we adopted 0.63 as a prior effect size. A power analysis was conducted based on the software developed by Westfall (2015, Unpublished). Given our sample size of 10 participants and 36 sentences per condition, the power of our present study is 0.93, a value which exceeds the minimum recommended level of 0.8 (Cohen, 1988).

### Apparatus

An SR EyeLink 2000 tracker with a 2000 Hz sampling rate was used to record participants’ right eye movements during reading. Each sentence was presented vertically in the centered of a 21 - inch CRT display monitor (DELL; resolution, 1024 by 768 pixels; frame rate, 120 Hz), with each character (font Song 20) position approximately equaling 0.9° of visual angle.

### Procedure

The experimenter seated the participant 70 cm away from the CRT display monitor. A chin rest and a head rest were used to minimize head movements. Prior to the experiment, participants were calibrated using a 3-point grid. Sentences were presented one by one after a successful calibration was completed. At the start of each sentence, a fixation point was presented on the left side of the computer screen. Participants were instructed to read each sentence silently, and then press a button to terminate the display and answer comprehension questions. About one-third of the total sentences consisted of yes–no comprehension questions. Fifteen sentences were read for practice before the formal experiment.

## Results

All of the participants in the analyses reported below scored at 95% accuracy or above on the comprehension questions, indicating that the participants read sentences carefully. We excluded the trials in which tracker loss occurred, as well as any first fixation durations that were shorter than 80 ms or longer than 1200 ms. In total, 5.54% of the data was excluded. In terms of the three regions of interests, 3.81% of the total data was excluded. In Region 1, 1.97% of the total data was removed. Across the four conditions, two types of structures (narrative-object biased structures and balanced structures) and two locations of prosodic boundary (after a verb and after “de”), the distribution of excluded data was 0.42, 0.56, 0.48, and 0.51%, respectively. In Region 2, 0.42% of the total data was removed. Across the four conditions, the distribution of excluded data was 0.07, 0.08, 0.13, and 0.14%, respectively. In Region 3, 1.42% of the total data was removed. Across the four conditions, the distribution of excluded data was 0.36, 0.19, 0.54, and 0.40%, respectively.

Three regions were selected as the regions of interest. Region 1 refers to the classifier phrase in the upstream context, which is composed of two characters (e.g., the phrase “
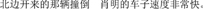
” in the example). This region can resolve the ambiguity of the “V+N1+de+N2” ambiguous phrase. Region 2 is the “V+N1+de+N2” ambiguous phrase itself (e.g., the ambiguous phrase “
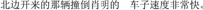
”” in the example). Region 3 contains the region immediately following the “V+N1+de+N2” ambiguous phrase, which is composed of two to three characters (e.g., the word “
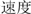
”” in the example). This region also serves to resolve the “V+N1+de+N2” ambiguous phrase.

For eye movement measures, first fixation duration (FFD, i.e., the duration of the first fixation on a region), gaze duration (GD, i.e., the sum of all fixations on a region before moving to another region), First-pass reading time (FPRT, a.k.a. gaze duration, which is used to analyze a region longer than a word, such as a phrase or a sentence), regression path duration (RPD, i.e., the total duration of all fixations from the first fixation in a region up to (but not including) the first fixation on a region to the right), total fixation duration (TFD, i.e., all fixation durations in a region) and the probability of regression out (REGP, i.e., the proportion of subjects making regression from a current region to previous parts) for each region were measured. FFD and GD are early measures of lexical processing that takes place when a region is fixated, whereas TFD provides an indication of the overall difficulty of a region caused by taking into account both the initial inspection and reinspection fixations (Inhoff, 1984; Rayner, 2009; Holmqvist et al., 2011). RPD and REGP for a region were the key measures representing reanalysis in reading (Liversedge et al., 1998; Clifton et al., 2003; Luo et al., 2015). Measures for all the three regions are summarized in Table 2.

**Table 2:** Means with standard errors of three regions under different conditions.

Measures	Prosodic boundary	Region 1		Region 2		Region 3	
				
		V-O bias	Balance	V-O bias	Balance	V-O bias	Balance
FFD	After “V”	220 (25)	219 (22)			261 (30)	256 (26)
	After “de”	206 (22)	208 (22)			257 (29)	254 (29)
GD/ FPRT	After “V”	257 (34)	250 (34)	768 (140)	776 (133)	330 (46)	323 (47)
	After “de”	233 (32)	229 (30)	810 (131)	758 (122)	334 (50)	308 (45)
TFD	After verb	706 (156)	579 (121)	2735 (507)	2178 (355)	725 (151)	615 (113)
	After “de”	576 (114)	553 (123)	2364 (359)	2121 (385)	699 (133)	629 (120)
REGP	After “V”	0.14 (0.08)	0.18 (0.12)	0.33 (0.14)	0.30 (0.14)	0.21 (0.12)	0.19 (0.11)
	After “de”	0.16 (0.10)	0.17 (0.12)	0.27 (0.13)	0.25 (0.11)	0.26 (0.15)	0.23 (0.13)
RPD	After “V”	351 (72)	372 (90)	1229 (199)	1210 (197)	866 (313)	693 (226)
	After “de”	351 (75)	347 (89)	1172 (192)	1115 (192)	805 (299)	649 (254)


Linear mixed models (LMMs) were run to determine the estimates for fixation duration and generalized linear mixed models (GLMMs) were used for REGP (Baayen et al., 2008). Both models adopt the lmer program of the lme4 package developed by Bates et al. (2019) in R 3.5.2 (R Core Team, 2018) and R Core Team (2018) for statistics calculation. Participants and items were treated as crossed random effects. The LMM and GLMM with the maximal random effects structure with both random intercepts and random slopes (Barr et al., 2013) was conducted. We always began with full models that included the maximum random effects structure. If the full model failed to converge, the random structure for items and the random effect correlations and the slopes were removed. *p-*values were calculated based on Satterthwaite’s approximations using the lmerTest package.

### Region 1

Region 1 is the most important region which can resolve the ambiguity of “V+N1+de+N2” ambiguous phrase. In this region, the main effects of prosodic boundary are found on FFD (*b* = -9.75, *SE* = 4.64, *t* = -2.10, *p* = 0.036) and GD (*b* = -18.23, *SE* = 6.75, *t* = -2.70, *p* = 0.006), suggesting that the FFD and GD are longer when prosodic boundaries occur after a verb than when they occur after “de.” Neither the main effect of the semantic bias type of ambiguous phrase nor the interaction between the semantic bias type of ambiguous phrase and prosodic boundary were significant on FFD and GD, *ts* < 1.

The main effects of the prosodic boundary (*b* = -80.10, *SE* = 24.56, *t* = -3.26, *p* = 0.001; *b* = -68.96, *SE* = 24.28, *t* = -2.84, *p* = 0.005) and the semantic bias type of ambiguous phrase (*b* = -73.41, *SE* = 24.55, *t* = -2.99, *p* = 0.003; *b* = -72.48, *SE* = 24.27, *t* = -2.99, *p* = 0.003) are found on TFD. Significant interactions between prosodic boundary and the semantic bias type of ambiguous phrase were found on TFD (*b* = 107.88, *SE* = 49.07, *t* = 2.20, *p* = 0.028). Further analysis showed that TFD is longer when prosodic boundaries occur after a verb than when they occur after “de” (*b* = -135.17, *SE* = 34.81, *t* = -3.88, *p* < 0.001), TFD of NOS - biased ambiguous structures are longer than balanced ambiguous structures (*b* = -128.37, *SE* = 34.78, *t* = -3.69, *p* < 0.001).

No main effects and interactions are present on REGP or RPD, *ts* < 1.

### Region 2

The region is the site of the experiment’s critical ambiguity. The main effect of prosodic boundary is found on REGP (*b* = -0.31, *SE* = 0.13, *z* = -2.48, *p* = 0.013) and RPD (*b* = -75.63, *SE* = 33.82, *t* = -2.24, *p* = 0.026), REGP and RPD are longer when prosodic boundaries occur after a verb than when they occur after “de.” Neither the main effect of the semantic bias type of ambiguous phrase nor the interaction between the semantic bias type of ambiguous phrase and prosodic boundary on REGP and RPD were significant, *ts* < 1.

The main effects of the prosodic boundary (*b* = -214.08, *SE* = 81.77, *t* = -2.62, *p* = 0.009) and the semantic bias type of ambiguous phrase (*b* = -400.66, *SE* = 81.77, *t* = -4.90, *p* < 0.001) are found on TFD. A marginally significant interaction between prosodic boundary and the semantic bias type of ambiguous phrase was found in this region on TFD (*b* = 314.29, *SE* = 163.54, *t* = 1.92, *p* = 0.055). Further analysis showed that TFD is longer when prosodic boundaries occur after a verb than when they occur after “de” (*b* = -371.22, *SE* = 116.61, *t* = -3.18, *p* = 0.001). Under the condition of NOS -biased ambiguous structures, TFD of NOS – biased ambiguous structures are longer than balanced ambiguous structures (*b* = -557.81, *SE* = 115.89, *t* = -4.81, *p* < 0.001).

No main effects and interactions are present on FPRT, *ts* < 1.

### Region 3

In region 3, the main effects of the semantic bias type of ambiguous structures are found on RPD (*b* = -171.93, *SE* = 58.68, *t* = -2.93, *p* = 0.003) and TFD (*b* = -89.56, *SE* = 24.10, *t* = -3.72, *p* < 0.001), RPD and TFD of NOS – biased ambiguous structures are longer than balanced ambiguous structures. There are no main effects of prosodic boundary and interactions with the semantic bias type of ambiguous structure on RPD and TFD, *ts* < 1.

No main effects and interactions are present on FFD, GD, and REGP, *ts* < 1.

## Discussion

By manipulating the location of prosodic boundary and semantic bias of “V+N1+de+N2” phrases, this study adopted the eye tracker to investigate the effect of prosodic boundary on ambiguity resolution in the two types of “V+N1+de+N2” ambiguous phrases, one being the narrative-noun biased structure and the other being the balanced ambiguous structure. We found that prosodic boundary had an influence on any related regions. Specifically, readers parsed ambiguous structures easier and faster when the prosodic boundaries occurred after “de” than when they occurred after “V.” When the prosodic boundary was positioned after “de,” the meaning conveyed by the ambiguous structure was compatible with the contexts, consequently promoting the processing of the ambiguous structure. In contrast, when the prosodic boundary was positioned after “V,” the meaning conveyed by the ambiguous structure was incompatible with the contexts, resulting in the increase of difficulty in processing. In this case, the reader spent more time reading and made more regressions, indicating they needed to integrate more cognitive sources to parse the sentence. With respect to time course of prosodic processing, we found prosodic boundary only influenced parsing at the later stage of sentence integration. More importantly, we found that the interaction between prosodic boundary and semantic bias occurred at the late stage of sentence processing.

This experiment found the main effect of prosodic boundary on FFD and GD, which reflect early reading time measures. However, it is highly possible that these increases in fixation duration were not triggered by prosodic information because the extracted prosodic information was employed only after the subjects fully completed processing the whole ambiguous structure, which was obviously out of the range of maximal visual angle field. Therefore, a conceivable explanation for the increase of FFD and GD could possibly be influenced by parafoveal preview information. According to previous research, readers can get information from 2° to 5° of visual angle field from fixation (Schotter et al., 2012). The blank space adopted as a prosodic boundary marker in this experiment was in the visual range of parafoveal information, therefore, this low-level visual clue can be obtained by the reader. When the prosodic boundary was placed after “V,” the blank space caused region 1 and the subsequent “V” to be processed as one chunk. Additionally, since the classifier phrase in Chinese commonly functions as the modifier of a noun rather than of a verb, Region 1 and the “V” combination is semantically incongruent which certainly increases the processing difficulty, thus leading to a noticeable increase of fixation in Region 1.

To further explore whether prosodic boundary played a role at the early processing stage, the processing data in Region 2 and Region 3 were analyzed. If prosodic boundary played a role at the earlier stage of sentence processing, we would expect to see an increase in the processing difficulty of FFD and GD or FPRT in these two regions. However, we found no significant differences on these two measures in Region 2 and Region 3, indicating the prosodic boundary played no role at the early stage of temporary ambiguous sentence processing. This result was inconsistent with previous studies which claimed the effect of prosody on ambiguous sentences during the early stage of reading (Traxler, 2009; Luo et al., 2015; Kentner and Vasishth, 2016), but consistent with some previous eye movement studies that reported no effect on FPRT (Kentner, 2012; Breen and Clifton, 2013). In particular, the findings were consistent with Yu and Yan (2015) which did not find the early effect of prosodic boundary on ambiguity resolution in the non-preferred context. Since the current study situated the “V+N1+de+N2 ambiguous structures” in the modifier-noun context, which in fact was the non-preferred context for narrative-object biased ambiguous construction, the non-preferred context might to some extent restrict the prosodic effect. Therefore, the current study also suggested that the effect of prosodic boundary at the early stage relies on the biased context.

Additionally, the fact that no prosodic effect was found at the early processing stage can be directly contributed to the characteristics of “V+N1+de+N2” ambiguous structure. The “V+N1+de+N2” structure involves complex phrasal ambiguity, and normally only consists of four words. In order to determine whether the prosodic boundary plays a role in disambiguation of this kind of structure, the subjects need to process the whole structure completely. Indeed, the subjects took a longer time reading because they were repeatedly parsing the ambiguous structure in an effort to determine the meaning. Therefore, it is almost impossible to find the prosodic boundary effect at the early stages of processing. The time differences found in the FPRT best illustrated this point.

The effect of prosodic boundary at the late stage of processing is undisputed. We observed the effect of prosodic boundary on TFD, which could reflect late stages in the comprehension process, in both Region 1 and Region 2.

We also found the effect of prosodic boundary on REGP and RPD on Region 2, strongly suggesting that when the meaning of the ambiguous structure guided by prosodic boundary is incongruent with the upstream context, more regressions toward the preceding regions were needed, which reflect the reader’s attempt to integrate the current information into the previous context (Bader, 1998; Frazier and Clifton, 1998; Liversedge et al., 1998; Boland and Blodgett, 2001).

One of the aims of this study was to explore the interaction between semantic bias and prosodic boundary. Of particular interest was examining whether semantic bias of ambiguous structures influence the effect of prosodic boundary. The study found no interaction between semantic bias and prosodic boundary at the early stage of processing. However, a noticeable interaction between these two was observed on TFD, which is an indicator of a relatively late effect on processing (Inhoff, 1984; Rayner, 2009), suggesting that prosodic effect is only influenced by semantic bias at the late stage where information integration is on-going. It is noteworthy that the effect of prosodic boundary was exhibited only in the narrative-noun biased ambiguous structures and not in the balanced ambiguous structure. In narrative-noun biased ambiguous structure, the prosody boundary presented the same effect in Region 1 and Region 2 pointing to the guiding function of implicit prosody in parsing. Prosodic boundary positioned after “V” was in line with the semantic bias of narrative-noun ambiguous structure motivating quick interpretation of narrative-noun meaning. However, the quickly obtained meaning was in conflict with the non-preferred modifier-noun context, which increased the difficulty in processing. As opposed to biased ambiguous structures, the balanced ambiguous structure has no semantic bias. The location of prosodic boundary, either after “V” or after “de,” activated the meaning of balanced structure not as greatly as it did in its narrative-noun biased counterparts which explains why prosodic boundary effect was found only in narrative-noun biased structure.

In the present study, we explored the role of prosodic boundary on ambiguity resolution during silent reading. Our findings were consistent with Li et al. (2010) and Yu (2011). Yu (2011) found that pause duration of prosodic boundary, as the most important prosodic clue of disambiguation, can dissolve “V+N1+de+N2” ambiguous structure. Li et al. (2010) used ERP to investigate the prosodic boundary effect during on-line syntactic processing. When the prosodic boundaries were incongruent with the syntactic interpretation, a left-anterior distributed LAN effect or a combined LAN and N400 effect was elicited, suggesting that prosodic information can be used in parsing the syntactic structure and can be immediately integrated with the ongoing discourse context in comprehending the spoken discourse. The current study also found that implicit prosody had a dominating effect on ambiguity resolution in “V+N1+de+N2” structures during silent reading. According to IPH, it is likely that prosody plays the same role in silent reading as it does in reading aloud. The current study shows that implict prosody plays an important role in parsing the ambiguous sturcture in silent reading just as overt prosody plays a crucial role in reading aloud (Fodor, 2002). Our study provided clear evidence that supports the IPH.

We examined the role of prosodic boundary in dissolving the ambiguity in the Chinese “V+N1+de+N2” structure which to date has been rarely studied. The “V+N1+de+N2” structure possesses different meaning and syntactic structure according to the different location of prosodic boundary. This ambiguous structure is quite different from ambiguous sentences in some western languages, where the modifier commonly occurs after the constituent it modified. While ambiguous structures differ in some aspects in different languages, the effect of implicit prosody on syntactic ambiguity resolution was found to be universal. This study provides evidence for the universal influence of IPH on syntactic parsing in silent reading of the “V+N1+de+N2” ambiguous structure in Chinese which has rarely been tested.

## Data Availability

All datasets generated for this study are included in the manuscript and/or the Supplementary Files.

## Ethics Statement

This study was carried out in accordance with the recommendations of the Human Research Ethics Committee of the Academy of Psychology and Behavior of Tianjin Normal University with written informed consent from all subjects. All subjects gave written informed consent in accordance with the Declaration of Helsinki. The protocol was approved by Human Research Ethics Committee of the Academy of Psychology and Behavior of Tianjin Normal University.

## Author Contributions

GY and MY conceived and designed the study. MY analyzed data of the study, wrote the manuscript. YY and BS wrote the manuscript.

## Conflict of Interest Statement

The authors declare that the research was conducted in the absence of any commercial or financial relationships that could be construed as a potential conflict of interest.
