# Supplementary material for: Effects of Implicit Prosody and Semantic Bias on the Resolution of Ambiguous Chinese Phrases
Source: Front Psychol. 2019 Jun 4;10:1308. doi: 10.3389/fpsyg.2019.01308 (PMC6558037; doi:10.3389/fpsyg.2019.01308)
Supplement: Supplementary file 4 [file Table_4.doc]

**Directions for Questionnaire on semantic bias of ambiguous structure**

Dear students,

The phrases presented in the table below are all ambiguous structures, for example，

咬 死 猎人 的 狗

bite to death hunter De dog

The above phrase can be interpreted as “咬死/猎人的狗”which is a modifier-head structure (MHS) that carries the meaning that the hunter’s dog was bitten to death. It can also be interpreted as “咬死猎人的/狗” which is a narrative-object structure (NOS) with the meaning that the dog bit and killed the hunter. Number 1 to 7 successively stands for the degree of semantic bias toward MHS to NOS. Number 4 stands for balanced structure(without semantic bias). Please judge the semantic bias of the following sentences based on your first intuition.

**Directions for rating of naturalness of the sentences**

Dear students, thanks for taking the time to participate in our experiment. Please rate the naturalness of the following sentences. Sentence naturalness in our research is defined as the extent to which a sentence is grammatically well-formed and natural according to native speakers’ intuition. Number 1 to 5 indicates the degree of naturalness of the sentences is gradually improved. Number 1 is the least natural, number 5 is the most natural, 3 is natural.

Please read each sentence carefully and mark“**△**”on the cell with the number you choose to rate.

Rating for naturalness is exemplified as below:

| **number** | **sentence** | **The least natural** | **Less natural** | **natural** | **More natural** | **The most natural** |
| --- | --- | --- | --- | --- | --- | --- |
| 1 | 教室里有五十多名外国学生。  There are over fifty foreign students in the classroom. | 1 | 2 | 3 | 4 | 5**△** |
| 2 | 有几只漂亮的蝴蝶翩翩起舞于花上。  Some beautiful butterflies are dancing gracefully above the flowers. | 1 | 2 | 3 | 4**△** | 5 |
| 3 | 我断定那人就是哥哥的王晓刚。  I am sure that the guy is my elder brother’s Wang Xiaogang. | 1**△** | 2 | 3 | 4 | 5 |

**Direction for rating of acceptablity of the sentences**

Dear students, thanks for taking the time to participate in our experiment. Please rate the acceptability of the following sentences. Sentence acceptablity is defined as the extent to which a sentence is semantically plausible according to the knowledge of the real world. Number 1 to 5 indicates the degree of acceptability of the sentences is gradually improved. Number 1 is the least acceptable, number 5 is the most acceptable, and 3 is acceptable.

Please read each sentence carefully and mark“△”on the cell with the number you choose to rate.

Rating for acceptability is exemplified as below:

| **number** | **sentence** | **The least acceptable** | **Less acceptable** | **acceptable** | **More acceptable** | **The most acceptable** |
| --- | --- | --- | --- | --- | --- | --- |
| 1 | 中午做饭时妈妈不小心切伤了西红柿。  When cooking at noon, mother accidentally cut and wounded the tomatoes. | 1**△** | 2 | 3 | 4 | 5 |
| 2 | 有几只漂亮的白蝴蝶翩翩起舞于花上。  Some beautiful white butterflies are dancing gracefully above the flowers. | 1 | 2 | 3 | 4 | 5**△** |
| 3 | 太阳每天从西边升起来从东边落下去。  The sun rises in the west and sets in the east every day. | 1**△** | 2 | 3 | 4 | 5 |
