# Supplementary material for: Effects of Implicit Prosody and Semantic Bias on the Resolution of Ambiguous Chinese Phrases
Source: Front Psychol. 2019 Jun 4;10:1308. doi: 10.3389/fpsyg.2019.01308 (PMC6558037; doi:10.3389/fpsyg.2019.01308)
Supplement: Supplementary file 5 [file Table_5.DOC]

**The experiment materials**

**Sentences that contain a narrative-object biased ambiguous phrases**

1. 我们看到那位*拜访教授的夫人*环顾书房，眼里充满敬意。

We noticed that the lady who visited the professor looked around the study room with eyes full of respect.

2. 邻居老张那个*拜会老舍的女儿*觉得老舍特别随和。

Laozhang’s daughter who humbly visited Laoshe thought Laoshe was very easy-going.

3. 老李觉得他那位*惩罚老叶的女婿*是有一定责任的。

Laoli holds that his son-in-law who punished Laoye is certainly responsible.

4. 我们公司那位*触犯孙刚的上司*后悔自己太冲动了。

The superior of our company who offended Sungang regrets acting out of impulse.

5. 记者发现那份*服从上级的决定*传达之后，大家很不满。

Everyone was upset after the journalist found that the decision to obey the superiors had been announced.

6. 交警发现那辆*回避元首的轿车*停在马路对面的教堂门口。

The traffic police found that the limousine that steered clear of the Head of State parked at the gate of the church opposite the street.

7. 大家都觉得那位*会见演员的替身*特别的紧张。

Everyone feels that the stand-in who will meet with the actors and actresses is very nervous.

8. 沙发上的那位*讥笑馆长的养女*认为不该总吃猪头肉。

The foster daughter on the couch who laughed at the curator thought people shouldn’t always eat pork head meat.

9. 李经理的那个*解雇高伟的侄子*认为自己的做法很正确。

The nephew who was fired by manager Li thought his behavior was quite acceptable.

10. 班长发现一座*靠近伪军的碉堡*上插一面日本旗。

The monitor found that there stood a Japanese national flag on the fort that is close to puppet army.

11. 上周来的那位*访问煤矿的英雄*穿着很朴素。

The hero who visited the coal mine last week was plainly dressed.

12. 乡里这位*心疼乡长的孙女*年龄还不满十六。

The granddaughter who loves the head of the township dearly is under sixteen.

13. 我们看到那位*询问汪川的管家*低声下气的。

We noticed that the steward who was inquiring about Wangchuan was timid and humble.

14. 李老师说所有*研究当代作家的著作*都没有解决他的疑问。

Teacher Li said all the works which study contemporary writers failed to answer his questions.

15. 我们注意到所有*拥护校长的提议*都认为校长为人公正。

We noticed that all the proposals that supported the schoolmaster regarded him as a man of integrity

16. 来自镇上的每位*援助山区的农民*都那么善良。

Every peasant from the town who carried aid to the mountain area was kind-hearted.

17. 新闻上说那位*照会各国的大使*认为战争可能一触即发。

The news reported that the ambassador who presented a diplomatic note to various countries thought it was likely that the war would break out at any moment.

18. 发布会上那些*指责报社的记者*都认为新闻报导必须客观。

All the journalists who criticized the newspaper office during the press conference thought that the news report must be objective and impartial.

19. 老李说那项*重视专家的建议*提交以后，引起强烈反响。

Laoli said that after the proposal that paid much attention to the experts’ suggestions was presented, it aroused an intense response.

20. 隔壁病房那位*嘱咐患者的家属*认为加强营养最重要。

The family member who was consoling the patient in the neighbor patient ward held that enhancing nutrition is most important.

21. 退伍老兵那些*寻找故人的足迹*遍布县城各条街道。

The old friends’ footprints for which the veteran looked were all over each street in the county.

22. 王成说那份影响大家的计划一旦得逞，后果不堪设想。

Wang Cheng said once the plan that influenced everyone succeeded, the consequences would be unimaginable.

23. 语言学院所有*了解中国的学生*都认为中国人非常勤奋。

All the students in the College of Linguistics who know a good deal about China hold that Chinese people are very diligent.

24. 最后那位*反对老张的爸爸*撤回了自己的最初决定。

The father which was last to oppose Laozhang withdrew his original decision.

25. 部长要求所有*欢迎外宾的代表*都必须着正装。

The minister required that all representatives who welcome foreign guest be formally dressed.

26. 报上说那支*击溃敌人的部队*受到上级嘉奖。

The newspaper said that the troop that utterly defeated the enemies was rewarded by the superiors.

27. 老李说那位*接待杨斌的朋友*态度十分友好。

Laoli said the friend who welcomed Yang Bin was very friendly and cordial.

28. 大家看到他那双*拉起妻子的双手*布满老茧。

Everyone noticed that his hands which were holding his wife hands were full of calluses.

29. 坐在老李旁边那个*惦记乔林的孩子*既懂事又可爱。

The child who was sitting close to Laoli and cared about Qiao Lin was not only sensible but also lovely.

30. 院长说那些*同意张华的意见*都来自于新入职的员工。

The dean said the opinions that supported Zhang Hua all came from new staff who had just been inducted into the company.

31. 小王说所有*批判作家的观点*都认为作家的思想有问题。

Xiaowang said all the opinions that criticized the writer thought that the writer had problems with his mind.

32. 小李说他那个*想念家乡的伙伴*很想回家看看。

Xiaoli said that the partner who missed his hometown wanted to go back home.

33. 老刘说那位*得罪王志的领导*脾气十分暴躁。

Laoliu said that the leader who offended Wang Zhi had a bad temper.

34. 妈妈说那位*等待孩子的老师*看起来很焦急。

Mother said the teacher who was waiting for the children looked very worried.

35. 目前一切*支持教授的观点*都认为转基因对身体有害。

At present, all opinions that support the professor think transgenesis is harmful to health.

36. 王娟说那位恐吓原告的律师将会受到起诉。

Wang Juan said that the lawyer who intimidated the accuser would be prosecuted.

**Sentences that contain balanced ambiguous phrases**

1. 突然冲出的那匹*保护小徐的战马*头部连中三枪。

The war horse that rushed out and protected Xiaoxu was hit by three shots to the head.

2. 商务部颁布的那项*抵制美国的政策*施行有半年了。

The policy issued by the commercial department that boycotted the USA had been enforced for about half of a year.

3. 刚进公司的那位*督促老吴的雇员*态度非常恶劣。

The staff member who just entered the company and hastened Laowu had a bad attitude.

4. 统计发现所有*告别山区的青年*都是八零后。

Statistics found that all the young who had bid farewell to the mountain area were born after 1980.

5. 病床前那位*护理丽丽的养父*坚信丽丽会好起来。

The foster father who was taking care of Lili had a strong belief that Lili would become better soon.

6. 历史已证明一切*反对人民的敌人*都将受到历史的惩罚。

History proves that all the enemies who are against the people will be punished by history itself.

7. 空军部队那位*教训老黄的参谋*后悔自己过于激动。

That adviser in the Air Force who taught Laohuang a lesson regretted his over-excited reaction.

8. 内科那个*接触小陈的医生*认为小陈没有大问题。

The doctor in the internal medicine department who contacted Xiaochen thought Xiaochen had no serious problems.

9. 邻居那只*惊动娟娟的小狗*满屋乱跑，样子很滑稽。

The neighbor’s little dog which startled Juanjuan ran around the house and looked very funny.

10. 球迷们认为所有*夸耀自己的球员*都没啥真正水平。

The ball fans held that all the players who boasted of themselves had no true potential.

11. 网上传出的那些*理解医院的呼声*来自各个领域的人士。

The voices from the internet that called for people’s understanding of hospitals came from public figures from various fields.

12. 一直讨论的那项*偏向老刘的方案*一共有80页。

The scheme favoring Laoliu which has been discussed all along contains 80 pages in total.

13. 刚刚进来的那位*劝说团长的副手*口齿十分伶俐。

The assistant who just came in and persuaded the commander was quick-witted.

14. 芳芳那副*讨厌处长的表情*流露一半又收回去了。

Fangfang’s emotional expression which showed disgust for the section chief was presented halfway and then retracted.

15. 一号展厅那些*欣赏国画的作者*都是有名的人物。

All the authors in Exhibit Hall No. 1 who were appreciating the traditional Chinese paintings were famous figures.

16. 老师说那个*帮助小张的同学*家里其实也不富裕。

The teacher said the classmate who helped Xiaozhang is actually not from a rich family either.

17. 小王觉得那个*喜欢阿姨的小孩*很像他儿子。

Xiaowang felt that the child who likes the aunt looks a lot like his son.

18. 打猎队伍里的那些*指望老袁的随从*情绪十分激动。

The hunting troops’ attendants who count on Laoyuan are quite excited emotionally.

19. 韩国球队那名*指责球员的陪练*认为谁也不能歪曲事实。

The training partners of the Korean football team who blamed the players thought that the truth could not be distorted by anyone.

20. 北边开来的那辆*撞倒肖明的车子*速度非常快。

The car that was coming from the north which ran into Xiao Ming was driving very fast.

21. 图书管里所有*介绍鲁迅的著作*都对鲁迅有很高的评价。

All the works in the library that introduce Luxun praise him highly.

22. 赵刚对那个*出卖自己的朋友*感到十分失望。

Zhao Guang was deeply disappointed in the friend who betrayed him.

23. 班长说那位*熟悉小张的连长*个子十分高大。

The monitor said that the captain who was very familiar with Xiaozhang was tall and strong.

24. 师长说两架*轰炸日军的战机*扭转了战争的被动局面。

The officer said that the two combat aircraft which bombarded the Japanese troops reversed the severe situation.

25. 战士们使用的三门*摧毁敌军的大炮*是从敌军手里夺来的。

The three cannons which were used by the soldiers to destroy the enemy were seized from the enemy.

26. 穿粉色衣服那个*打扮新娘的伴娘*觉得化妆不宜太浓。

The bridesmaid who was dressed in pink and making up the bride thought that the makeup shouldn’t be too thick.

27. 我们看到那位*回访北大的书记*笑容满面，十分亢奋。

We noticed that the secretary who paid a return visit to Beijing University beamed with a smile and was very excited.

28. 书记说那些*保护工厂的职工*都是即将入党的积极分子。

The secretary said the staff that protected the factory were all activists who would soon join the party.

29. 院长说这两篇*赞扬小王的文章*都是由敬老院的老人写的。

The dean said the two papers which praised Xiaowang were written by the elderly from the nursing home.

30. 报上说那位*歌颂祖国的劳模*认为荣誉是群众给予他的。

The newspaper said the model worker who praised the motherland thought the reputation had been bestowed upon him by the masses.

31. 我们看见那位*回避张嫂的表姐*偷偷溜进厕所。

We saw the cousin who avoided Zhangsao secretly slip into the restroom.

32. 小张觉得那个*笑话小孙的室友*品质极其恶劣。

Xiaozhang thought the roommate who laughed at Xiaosun was a person of low quality.

33. 小王的那位*刁难小赵的弟弟*性格十分古怪。

Xiaowang’s younger brother who deliberately made things difficult for Xiaozhao was very weird.

34. 张姐反映所有*扰乱社区的居民*都是素质极其低劣的人。

Sister Zhang reported that all the citizens who disturbed the community were people of low quality.

35. 晚报上那则*封锁港口的消息*一出立刻引起渔民们的轰动。

As soon as the news about the blockade of the port was published in the evening newspaper, it created a stir among the fishermen.

36. 小张说那位*乞求哥哥的朋友*说话真是啰嗦。

Xiaozhang said the friend who begged his brother spoke in a long-winded way.
